# Enhanced blood–brain barrier penetration and glioma therapy mediated by T7 peptide-modified low-density lipoprotein particles

**DOI:** 10.1080/10717544.2018.1494223

**Published:** 2018-11-03

**Authors:** Meng Liang, Chunhong Gao, Yuli Wang, Wei Gong, Shiyao Fu, Lin Cui, Zhenhan Zhou, Xiaoyang Chu, Yue Zhang, Qianqian Liu, Xiong Zhao, Baoquan Zhao, Meiyan Yang, Zhiping Li, Chunrong Yang, Xiangyang Xie, Yang Yang, Chunsheng Gao

**Affiliations:** a State Key Laboratory of Toxicology and Medical Countermeasures, Beijing Institute of Pharmacology and Toxicology, Beijing, China;; b Department of Pharmacy, Wuhan General Hospital of the PLA, Wuhan, China;; c Department of Pharmacy, Jiamusi University, Jiamusi, China;; d 307 Hospital of the PLA, Beijing, China;; e Beijing Institute of Health Service and Transfusion Medicine, Beijing, China

**Keywords:** Low-density lipoprotein particles, T7 peptide, brain-targeted drug delivery, glioma, vincristine

## Abstract

Therapeutic outcome for the treatment of glioma was often limited due to the non-targeted nature and low permeability of drugs across the blood-brain barrier (BBB). An ideal glioma-targeted delivery system need to traverse the BBB and then target glioma cells with adequate optimized physiochemical properties and biocompatibility. However, it is an enormous challenge to the researchers to engineer the above-mentioned features into a single nanocarrier particle. New frontiers in nanomedicine are advancing the research of new biomaterials. In this study, we demonstrate a strategy for glioma targeting by encapsulating vincristine sulfate (VCR) into a naturally available low-density lipoprotein particles (LDL)-based drug delivery system with the modification of T7 peptide ligand (T7-LDL). LDL, endogenous lipid transporters, can specifically bind to brain endothelial cells and glioma cells via interacting with the low-density lipoprotein receptors (LDLR). T7 is a seven-peptide ligand of transferrin receptors (TfR) capable of circumventing the BBB and then targeting glioma. By combining the dual-targeting delivery effect of T7 peptide and parent LDL, T7-LDL displayed higher glioma localization than that of parent LDL. After loading with VCR, T7-LDL showed the most favorable antiglioma effect *in vitro* and *in vivo.* These results demonstrated that T7-LDL is an important potential drug delivery system for glioma-targeted therapy.

## Introduction

Glioma is considered as one of the most aggressive and lethal types of cancers. The clinical therapeutic effect of glioma by drug treatment is very unsatisfying because of the existence of the blood–brain barrier (BBB), which restricts the distribution of drugs from blood to brain (Pardridge, 1999). Moreover, the non-targeted nature of drugs often causes severe side effects because they produce a similar cytotoxicity in both cancerous and healthy cell. Given the intrinsic difficulty for most conventional drugs to reach the brain, the use of nanocarriers has been suggested as an innovative therapeutic strategy (Kreuter, 2001). Over the past few decades, various types of nanocarriers such as liposomes, mesoporous silica nanoparticles, carbon nanotubes, and polymeric particles have been fabricated from various materials (Shaw et al., 2017). In recent years, to overcome the BBB barrier and specifically deliver drugs to brain tumor, dual targeting delivery strategy have been extensively explored (Gao, et al., 2013; Gao, 2016; 2017), which are functionalized with two active targeting ligands: one to the BBB and the other to the brain tumor. There are many targeting ligands that have been deeply studied, including TGN peptide, AS1411 aptamer (Gao et al., 2012), interleukin-13 peptide, RGD (Gao et al., 2014), angiopep-2 (Ruan et al., 2015), Ala-Ala-Asn-Cys-Asp (AK) peptide (Ruan et al., 2017), and so on. Nanocarriers have several advantages in drug delivery: high payload of drugs, prolonging blood circulation time, targeting specific cells, and enhancement of endocytosis (Qu et al., 2016). However, issues related to their synthetic materials (e.g. porous hollow silica or not fully biocompatible polymers) can limit their effects and cause toxicology problems (Nativo et al., 2008; Guerrero-Cázares et al., 2014).

To circumvent the obstacles imposed by nanocarriers that consist of synthetic materials, suitable nanocarriers should be developed using natural materials, which are considered nontoxic for the organism and thus safe (Moghimi et al., 2005; Symonds et al., 2005; Gao et al., 2011). In recent years, several natural materials have emerged as nanocarriers to deliver drug to treat diseases in the clinic or preclinical trials, such as paclitaxel albumin-bound particles (Abraxane^®^). However, because of a lack of specific targeting, it may be difficult for some natural nanocarriers to accumulate in brain merely based on their nanosize. Fortunately, the BBB cannot be intact. The brain is dependent on the blood to deliver metabolic substrates and remove metabolic waste, and the BBB therefore facilitates the exchange of selected solutes. In recent years, many endogenous transporters that facilitate the uptake of nutrients and minerals have been revealed in the cerebral endothelium (Chen et al., 2017). Lipoproteins are endogenous lipid transporters within the circulation that shuttle cholesterol and triglycerides between various tissues in the body. Among these lipoproteins, low-density lipoprotein (LDL) has attracted tremendous attention for its application in cargo loading and delivery owing to its unique features. (1) Biosafety: LDL is the major transporter of cholesterol in humans and therefore is biocompatible and nonimmunoqenic. (2) Nanostructure: Unlike liposomes, for example, LDL particles are solid particles that consequently have greater structural stability than liposomes (Chu et al., 2001). (3) Targeting: LDL is the major ligand for the low-density lipoprotein receptors (LDLR) (Zhu et al., 2017). It can cross the BBB with LDLR and deliver cholesterol to the brain. Interestingly, because LDLR is both highly expressed in BBB cells and glioma cells, it is an ideal target for glioma-targeted drug delivery (Burdo & Connor, 2003). Therefore, these features and observations inspired the current investigation of looking into the potential of LDL as a carrier system to deliver cargo selectively to glioma cells.

Although LDL is a useful vehicle for lipophilic drugs, its application is largely limited because of low entrapment efficiency (Zhu et al., 2014). So far, most of published works on LDL-based brain-targeted delivery system only reported *in vitro* results. In order to meet this challenge, Mulik et al. reported a strategy that localized delivery of drug to the brain is possible using systemically administered LDL nanoparticles combined with pulsed focused ultrasound exposures in the brain (Mulik et al., 2016). However, reversible opening of the BBB by ultrasound may leave the brain parenchyma susceptible to accumulating deleterious compounds from blood. Therefore, a major component of LDL research is ‘targeting’, which is addressed by surface conjugation of LDL with ligands (e.g. peptide and antibodies) that can efficiently target the diseased sites. Compared with other nanocarriers, LDL carrys reactive groups (e.g. thiol, amino, and carboxylic groups) on its surfaces that can be used for ligand binding by covalent linkage. To enable the LDL-based glioma-targeted delivery system to target both the BBB and glioma, the ligand needs to be recognized by both. Transferrin receptors (TfR) has been observed to express on both the BBB and glioma cells (Kang et al., 2015). Thus, the corresponding ligand could be used for the delivery system to the BBB and glioma cells. A seven-peptide (sequenced HAIYPRH, T7) screened by a phage display system has a higher affinity for TfR, with a K_d_ of ∼10 nM. In recent years, T7 peptide has been used as a ligand in glioma-targeted drug delivery systems (Shinde & Devarajan, 2017; Zhang et al., 2017). Thus, we used T7 in this study to enhance the LDL to penetrate through the BBB and actively target glioma. T7 modified LDL (abbreviated as T7-LDL) can efficiently cross the BBB and bind to glioma cells via interacting with the TfR and LDLR. This design is intended to improve the selective delivery to the BBB and glioma cells, and to reduce intrinsic toxicity to healthy cells beyond the reliance upon the EPR effect and mono-targeting modification.

Vinca alkaloid vincristine (VCR) has also been widely used as a broad-spectrum antitumor drug since the 1960s, mainly for lymphoma and leukemia. Although VCR inhibited the proliferation of glioma cells *in vitro*, it failed to show any benefit in the treatment of glioma patients, which was ascribed to the poor glioma-targeting ability and restriction provided by the BBB. Therefore, we used VCR as a model drug to evaluate the glioma-targeting ability and treatment efficiency of the constructed delivery system.

In this study, *in vitro* and *in vivo* experiments were performed to explore the targeting delivery effect of T7-LDL. VCR was loaded into the particles to evaluate the antiglioma effect of T7-LDL. Herein, we report the first study on peptide-modified LDL as a glioma-targeted delivery system. The findings have provided valuable preclinical data to validate a noninvasive, efficient targeted peptide-nanotherapy for treatment of glioma, one of the most untreatable and deadly malignant diseases.

## Experimental materials

### Materials

Human plasma was obtained from Beijing Institute of Transfusion Medicine (Beijing, China). Sulfate vincristine (VCR) was obtained from Baiyunshan Co. (Guangzhou, China). T7 with a cysteine on the N-terminal (Cys-T7) was synthesized by Cybertron medical technology Co. (Beijing, China). All chemicals were of reagent grade and were obtained from Sigma-Aldrich, unless otherwise stated. Glioma C6 cells and mouse brain endothelial bEnd.3 cells were provided by the Cell Resource Centre of IBMS (Beijing, China) and cultured in Dulbecco’s modified Eagle’s medium (DMEM) containing 10% FBS (Gibco, Carlsbad, CA).

Female ICR mice (weighing 22–24 g) were purchased from Vital River Laboratories (Beijing, China). All animals were handled according to the code of ethics in research, training, and testing of drugs as laid down by the Animal Care and Use Ethics Committee of Academy of Military Medical Sciences.

### Methods

#### Isolation of LDL

LDL was isolated from human plasma by density gradient ultracentrifugation as described before (Zhu et al., 2014) and characterized by infrared spectrum and ultraviolet visible spectrum. LDL was finally stored at 4 °C until further use within 2 weeks.

### Preparation of VCR-loaded LDL

The VCR-loaded LDL was prepared by direct hydration of a lipid film. Briefly, 15 mg of VCR or hydrophobic probe (Cy5.5) was dissolved with chloroform in a pear-shaped flask and were subsequently evaporated to form dry film using a rotary evaporator under vacuum. The lipid film was then hydrated using PBS containing 120 mg of LDL at 37 °C for 24 h. To control for the size, the lipid dispersion was extruded 11 times through 100 nm polycarbonate filters using a mini extruder (Avanti, Canada).

### Preparation of T7-LDL

The preparation of T7-modified LDL (T7-LDL) consisted of two steps. First, NHS-PEG_3500_-T7 was synthesized by conjugating NHS-PEG_3500_-Mal with Cys-T7. Briefly, NHS-PEG_3500_-Mal (3 μmol) and Cys-T7 (4.5 μmol) were dissolved in phosphate buffer (pH 8.0) at room temperature for 24 h while stirring. After thin-layer chromatography (TLC) showed the disappearance of NHS-PEG_3500_-Mal, the reaction mixture was dialyzed (molecular weight cutoff (MWCO) 300 kDa, Thermo Scientific, Waltham, MA) in distilled water for 48 h to remove the unreacted Cys-T7. After the lyophilization step, NHS-PEG_3500_-T7 was obtained and confirmed by MALDI-TOF MS. Secondly, T7-LDL was prepared by coupling NHS-PEG_3500_-T7 to VCR-loaded LDL or Cy5.5-labeled LDL through their amino group as follows: amount of NHS-PEG_3500_-T7 (0.5%, 1%, 2%, 3%, 4%, and 6%, molar ratio) was reacted with Cy5.5-labeled LDL in PBS (pH 7.4) at room temperature with stirring for about 24 h, respectively. For VCR-loaded T7-LDL, the content of conjugates was 4%. The final solution was lyophilized and stored at −20 °C until further use.

### Connection efficiency of T7 peptide onto LDL nanoparticles

#### Fluorescence probe labeling and characterization

The carboxyl group in fluorescence probe (5-(6)-carboxyfluorescein diacetate, CFDA) was reacted with the primary amine group in the T7 of NHS-PEG_3500_-T7 and formed the target product NHS-PEG_3500_-T7(CFDA). Briefly, 5 mg of CFDA was dissolved in 1 mL of dimethyl sulfoxide, followed by adding 2.4 mg of N,N′-dicyclohexyl carbodiimide and 1.3 mg of NHS. This system was stirred for 24 h at room temperature, and then the insoluble substances were removed by centrifugation (4000 r·min^−1^, 15 min) from it. After that, 2 mg of NHS-PEG_3500_-T7 and 1 μL of triethylamine were added to the supernatant at room temperature, and reacted for 24 h avoiding light. Then, the above system was dialyzed (MWCO 3.5 kDa, Thermo Scientific, Waltham, MA) in distilled water without light for 24 h to remove the unreacted products. The dialyzed products (NHS-PEG_3500_-T7(CFDA)) were freeze-dried and obtained puffed solid products, which were finally characterized by MALDI-TOF MS.

The CFDA-labeled T7-modified LDL (T7(CFDA)-LDL) was prepared followed the same procedures of preparation of T7-LDL, except the NHS-PEG_3500_-T7 was partially substituted by NHS-PEG_3500_-T7(CFDA) (4%, molar ratio).

#### UV spectrum scanning

The PBS (pH 7.4) dispersion system was prepared by proper amount of VCR solution, NHS-PEG_3500_-T7 (CFDA) or LDL suspension, respectively. The UV visible spectrophotometer was used to scan the spectrum in 200–600 nm to determine the maximum absorption wavelength of NHS-PEG_3500_-T7 (CFDA). Standard solutions of CFDA was prepared (1.50, 2.00, 2.50, 3.00, and 3.50 μg·mL^−1^) and their absorbance (*A*) at 493 nm was measured by ultraviolet visible spectrophotometer. Then, the UV absorbance (*A*) and concentration (*C*) were used to perform the linear regression analysis.

### Characterization of nanocarriers

The mean diameter and particle distribution of these nanocarriers were measured by dynamic light scattering (Nanophox, Sympatec GmbH, Germany). Morphology of the VCR-loaded T7-LDL was characterized via a transmission electron microscopy (TEM) (HITACHI, H-7650, Japan), respectively. The stability of VCR-loaded T7-LDL in full rat serum was evaluated using a Turbiscan Lab^®^ Expert (Formulaction, L’Union, France). The analysis of stability was carried out by the software of the instrument, as a variation of back-scattering (ΔBS) profiles.

The VCR encapsulation efficiency (EE) of VCR-loaded T7-LDL and VCR-loaded LDL was calculated using the following equation:

$$\text{EE}\%=\left(W_{\text{total}\;\text{drug}}-W_{\text{free}\;\text{drug}}\right)/W_{\text{total}\;\text{drug}}\times 100\%$$

Where *W*_total drug_ and *W*_free drug_ represent the total drug in nanocarriers and the amount of free drug in the ultrafiltrate, respectively.

#### Samples analysis

The T7 (CFDA)-LDL were diluted 10 times with PBS, and the total absorbency (*A_Total_*) was measured at the maximum absorption wavelength of CFDA. Then, the above samples were put into the ultra-filtration centrifuge tube (MWCO 300 kDa) to centrifuge (8000 r·min^−1^) for 15 min and collected the sublayer liquids. The sublayer liquids were diluted four times and their absorbency (*A_Free_*) was measured. The connection efficiency of T7(CFDA)-LDL was calculated as following:

$$\text{Connection}\;\text{efficiency}=(10A_{\text{Total}}\;-\;4A_{\text{Free}})/10A_{\text{Total}}\times 100\%$$

### Cellular uptake

In order to assess the binding affinity of different nanocarriers to cells, different 5 μM Cy5.5-labeled T7-LDL or Cy5.5-labeled LDL were incubated with two types of cells (C6 cells and bEnd.3 cells) at 37 °C for 2 h, respectively. The cells were washed three times with cold PBS, then centrifuged and resuspended with PBS for qualitative analysis by confocal laser scanning microscopy (CLMS) (UltraVIEW Vox, PerkinElmer, Waltham, MA).

### Effect of peptide density on crossing *in vitro* BBB model

An *in vitro* BBB model was constructed to estimate the penetration efficiency of T7-LDL in mimicking conditions *in vivo*. To establish *in vitro* BBB model, a bEnd.3/C6 co-culture BBB model was established according to previous reports (Chen et al., 2017). Briefly, bEnd.3 cells were seeded on the upper side at 1 × 10^5^ cells per insert (Corning, NY). C6 cells were seeded on the basolateral compartment of the insert at 5 × 10^5^ cells/compartment. The cell monolayer integrity was measured by transendothelial electrical resistance (TEER). *In vitro* BBB model was considered constructed successfully when the TEER value reached 200 Ω·cm^2^.

To investigate the effect of T7 peptides density on cellular uptake after penetrating the *in vitro* BBB model, Cy5.5-labeled T7-LDL was prepared at different peptide densities (0%, 0.5%, 1%, 2%, 3%, 4%, and 6%, molar ratio). Various Cy5.5-labeled nanocarriers were added to the apical compartment of the *in vitro* BBB model. The bEnd.3 cells on the upper side were incubated with different formulations for 4 h at 37 °C and the C6 cells on the basolateral compartment were rinsed with cold PBS, trypsinized, and washed three times with cold PBS. The samples were then centrifuged and resuspended with PBS. Approximately 10^5^ C6 cells were applied immediately using a flow cytometry (FCM) (BD FACSCalibur, Franklin Lakes, NJ). The final concentration of Cy5.5 was 150 ng·mL^−1^.

### *In vitro* of VCR release

Dialysis was performed to investigate the *in vitro* release of VCR from the VCR-loaded T7-LDL. The release medium was PBS buffer (0.1 M, pH 7.4). About 2 mL of VCR-loaded T7-LDL or VCR-loaded LDL was added to dialysis bag with molecular weight cut off 12,000–14,000. The dialysis bag was then placed in a flask filled with 30 mL medium at 37 °C. At predetermined intervals, 800 μL of medium was drawn out and replenished with the same volume of fresh medium. The released free VCR at different incubation times was assayed by HPLC, as previously reported (Li et al., 2016).

### Cytotoxicity

C6 cells were seeded into 96-well plates at the density of 5000 cells/well and incubated for 24 h at 37 °C. Then, cells were treated with various samples (blank LDL, blank T7-LDL, free VCR, VCR-loaded T7-LDL, and VCR-loaded LDL) at a range of concentrations. The cytotoxicity of each sample was determined by MTT method in triplicate.

### Brain targeting ability of T7-LDL in zebrafish

Zebrafish were incubated according to a report (Lal et al., 2012). The fish were divided into groups, and 10 nL of free Cy5.5, Cy5.5-labeled T7-LDL, or Cy5.5-labeled LDL (0.1 mg·mL^−1^) was injected into the heart of zebrafish by a micro-sprinkler, respectively. After 10 min, the brain of fish was visualized and imaged by a CLSM (100× magnification, 673 nm for the excitation wavelength).

### Glioma targeting ability of T7-LDL in mice with intracranial glioma

Glioma-bearing mice model was established as our research team previously reported (Gong et al., 2011). The intracranial glioma-bearing mice were administered to Cy5.5-labeled T7-LDL or Cy5.5-labeled LDL (diluted to 0.2 ml by physiological saline) via tail vein injection. Four hours after the i.v. injection, mice were sacrificed, and the brain and main organs were dissected for *ex vivo* imaging by an IVIS^®^ Spectrum (Caliper, Alameda, CA). Fluorescent signals were quantified using Living Image^®^ software (Caliper, Alameda, CA). Immunofluorescence assay was performed by injecting Cy5.5-labeled T7-LDL or Cy5.5-labeled LDL (0.5 mg/kg, diluted to 0.1 ml by physiological saline). After 4 h, the intracranial glioma-bearing mice were anesthetized, and the hearts were perfused with saline, which was followed by 4% paraformaldehyde. The brains were removed for consecutively preparing 5-μm-thick frozen sections. Nuclei were stained with DAPI. The distribution of fluorescence was observed using CLSM.

### *In vivo* antiglioma effect

The intracranial glioma-bearing mice were randomly divided into the following four groups (10 mice per group): Free VCR group, VCR-loaded T7-LDL group and VCR-loaded LDL group, and physiological saline group. Eight days after cell injections, each mouse received a dose of 1 mg/kg four times every 2 days. At day 16, four mice from each group were anesthetized and brain cancer was assessed by magnetic resonance imaging (MRI) (Siemens, Munich, Germany) with measurement of the tumor diameter. Glioma inhibition was calculated using the formula: *R*_v_ = (*V*_drug_/*V*_saline_) × 100%. Where *V*_drug_ is the glioma volume after treatment with drug and *V*_saline_ is the glioma volume after treatment with physiological saline. The remaining six mice in each group were used to monitor survival. The survival time was calculated from day 0 (tumor inoculation) to the day of death. Kaplan–Meier survival curves were plotted for each group. Meanwhile, the body weight of each mouse was measured daily.

### Histology of brain tumors

After the treatments were finished, the mice were sacrificed to collect the brains. Brain tumors were fixed in 10% buffered formalin, embedded in paraffin, and sectioned at 5 μm thickness. Sections were stained with Hematoxylin and Eosin (HE). The tumor histology was viewed and imaged under optical microscopy (Olympus Company, Tokyo, Japan).

### Statistical analysis

The data are presented as the means ± standard deviation (SD). The difference between any two groups was determined via ANOVA. *p* < .05 was considered to be statistically significant.

## Results and discussion

### Identification of nanocarriers

Following the density gradient ultracentrifugation, LDL in the third layer (Figure S1) was collected (1.019–1.063 g·mL^−1^). Infrared spectrum study was performed to verify the isolation of LDL. As shown in Figure S2, the weak absorption peak at 3282.8 cm^−1^ was the –OH stretching vibration peak of water molecules. The peaks at 2925 and 2852 cm^−1^ were symmetric and asymmetric expansion vibrations of the fat chains of LDL. The stretching vibration peaks of C=O from phosphatidylcholine chain, and peptide were at 1736 cm^−1^ and 1653 cm^−1^, respectively. The peak at 1241 cm^−1^ was the stretching vibration of P = O. The peak at 1088 cm^−1^ was the balanced stretching vibration of PO_2_−, and the 969 cm^−1^ peak was the asymmetric vibration peak of the choline aggregates N^+^(CH_3_)_3_ in the phospholipid. These characteristic peaks were consistent with the previous report (Iwanik et al., 1984). These results suggested that the method adopted in this study was suitable for the isolation of LDL from human blood.

**Figure 1.**
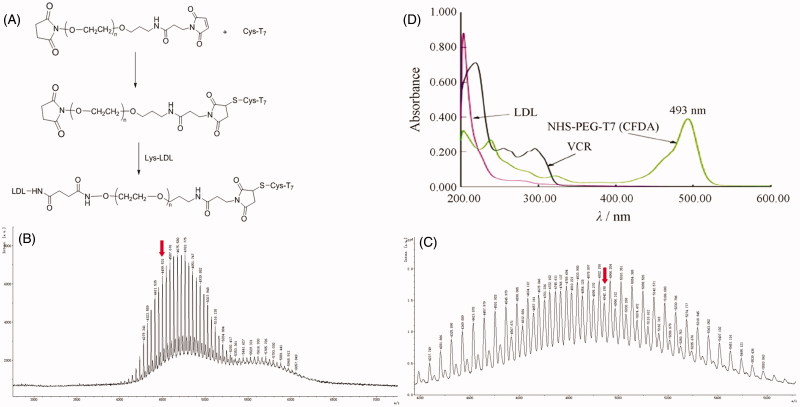
(A) Principle of the preparation of T7-LDL. MALDI-TOF mass spectra of (B) NHS-PEG2000-T7 and (C) NHS-PEG2000-T7(CFDA). Ultraviolet visible spectrum scan of (D) LDL, NHS-PEG2000-T7(CFDA) and free VCR solution. Red arrows represent the theoretical mass-charge ratios of (B) NHS-PEG2000-T7 and (C) NHS-PEG2000-T7(CFDA).

In an effort to enhance BBB penetration and glioma targeting of LDL, it was functionalized with T7 peptide in this study. The preparation scheme of T7-LDL is illustrated in Figure 1(A). First, NHS-PEG_3500_-T7 was synthesized. The T7 was terminated with cysteine to introduce free sulfhydryl (–SH), and this material was conjugated to NHS-PEG_3500_-Mal via the sulfhydryl-maleimide reaction, which enabled T7 to be conjugated at a specific site (–SH). The MALDI-TOF MS results confirmed the successful formation of NHS-PEG_3500_-T7, with the observed mass-charge ratios of approximately 4499.531 (Figure 1(B), marked by an arrow), which was equal to the theoretical mass–charge ratios of 4499. Secondly, T7-LDL was prepared by coupling NHS-PEG_3500_-T7 to LDL through *N*-hydrosuccinimide-amino coupling reaction. It is now still a great challenge to quantitatively measure the connection efficiency of modified ligands that are successfully attached onto the nanocarriers, especially for peptides, proteins, and other biological macromolecules for targeting purposes.

In this part, we designed an indirect but simple methodology to determine the connection efficiency of T7 peptide onto the LDL nanoparticles. As the MALDI-TOF MS results revealed, the molecular mass of NHS-PEG_3500_-T7(CFDA) (Figure 1(C)) was 4942.198. The theoretical molecular mass of NHS-PEG_3500_-T7(CFDA) was 4943. The MALDI-TOF MS results confirmed that NHS-PEG_3500_-T7(CFDA) was successfully synthesized. It should be noted that the difference between the ion peaks in Figure 1(B) and (C) is 44 units, which represented a repeat unit –CH_2_CH_2_O– in the structure of the PEG. As shown in Figure 1(D), the absorption peak of NHS-PEG_3500_-T7(CFDA) at 493 nm has no interference with LDL and VCR. Therefore, 493 nm was chosen to measure the connection efficiency of prepared modified nanocarriers. The linear regression equation of CFDA was *A* = 0.1950*C* + 0.0071 (*R* ^2^ = 0.9993, *n* = 5), the concentration ranged from 1.5 to 3.5 μg·mL^−1^. According to this equation, the final connection efficiency of T7 on the prepared nanoparticles was 63.88%.

### Characterization of T7-LDL

**Figure 2.**
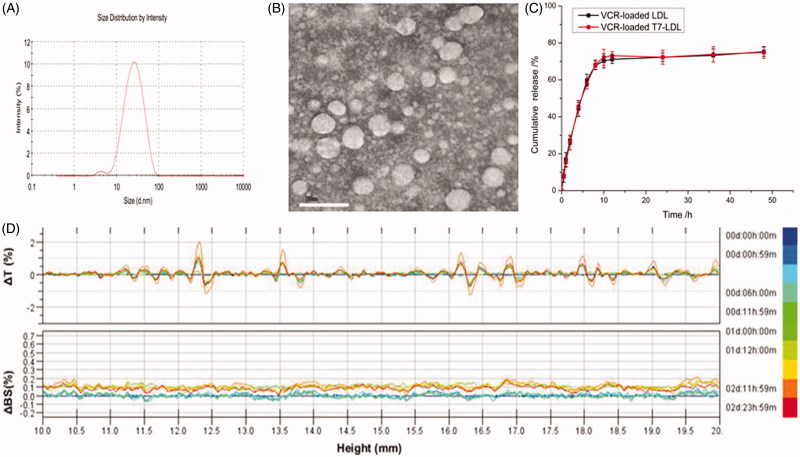
Physicochemical characterization of VCR-loaded T7-LDL. (A) Particle size distribution of VCR-loaded T7-LDL. (B) Morphological appearance of VCR-loaded T7-LDL based on TEM. Stability of VCR-loaded GKRK-APO in the full rat serum. (C) The transmission and backscattering profiles were measured at each time point using a Turbiscan Lab^®^ Expert analyzer. (D) *In vitro* release of VCR from T7-LDL and LDL at pH 7.4 at 37 °C, respectively. The data are presented as the means ± SD (*n* = 3). *Indicates *p* < .05.

The physico-chemical properties of VCR-loaded T7-LDL and VCR-loaded LDL are summarized in Table 1. The VCR encapsulation efficiency (EE) of the two distinct nanocarriers was more than 30%, and the modifications of T7 on the surfaces of the LDL did not affect the ultimate encapsulation efficiency. For an ideal nanocarrier, nanoparticle size would be a precondition and a crucial factor which decided the fate of nanocarrier both *in vivo* and *in vitro*. After EE study, the particle size of VCR-loaded T7-LDL was further analyzed by laser particle analyzer. The mean particle size of the VCR-loaded T7-LDL was 30.26 ± 2.21 nm, and it had a narrow size distribution (the polydispersity index was 0.0368 ± 0.0023) (Figure 2(A)). This particle size was suitable for delivery in the circulation because this size was sufficiently small to cross into tissues, approach cell surface receptors and facilitate intracellular transport (Zhao et al., 2012). TEM (Figure 2(B)) observation confirmed the results of laser particle analyzer. The VCR-loaded T7-LDL was monodispersed in solution with a well-defined spherical morphology. The VCR-loaded T7-LDL stability against physiological conditions is a prerequisite for further application *in vivo*, and thus, full rat serum was employed to mimic the *in vivo* situation. The *in vitro* stability of the VCR-loaded T7-LDL and VCR-loaded LDL in the full rat serum was evaluated using Turbiscan Lab^®^ Expert. According to this judgment (Celia et al., 2009), the transmission or back-scattering profiles (less than 0.5%) obtained (Figure 2(C)) indicating there was no apparent aggregation or sedimentation occurred of VCR-loaded T7-LDL in the culture medium during 72 h. The *in vitro* VCR release study was also performed to examine the drug release property of the T7-LDL. As shown in Figure 2(D), there were no pronounced differences in VCR release behavior between the two types of nanocarriers at each time point. The similar physicochemical characteristics of T7-LDL and LDL allowed us to specifically compare the effects of ligand modification on the LDL uptake and anticancer abilities.

**Table 1.** Characteristics of the nanocarriers.

Sample ID	Diameter (nm)	Polydispersity index	Encapsulation efficiency (%)	Drug loading efficiency (%)	Zeta potential (mv)
VCR-loaded LDL	28.88 ± 3.27	0.0315 ± 0.0014	30.41 ± 0.11	3.76 ± 0.08	−5.91 ± 0.32
VCR-loaded T7-LDL	30.26 ± 2.21	0.0368 ± 0.0023	30.12 ± 0.09	3.72 ± 0.09	−11.52 ± 0.87

The data are expressed as the mean ± SD for three different preparations (*n* = 3).

### Binding of different nanocarriers to cells

To determine whether the affinity of LDL to cells exhibits a difference after modification, Cy5.5-labeled T7-LDL or Cy5.5-labeled LDL was incubated with bEnd.3 and C6 cells at 37 °C for 2 h, respectively. bEnd.3 cells, the main component of the BBB, was selected as the TfR and LDLR-positive cell type used to investigate the brain targeting ability of nanocarriers (Hu et al., 2009). C6 cells overexpressing TfR and LDLR were used as the models of glioma to confirm the glioma targeting ability of nanocarriers (Zhang et al., 2013).

**Figure 3.**
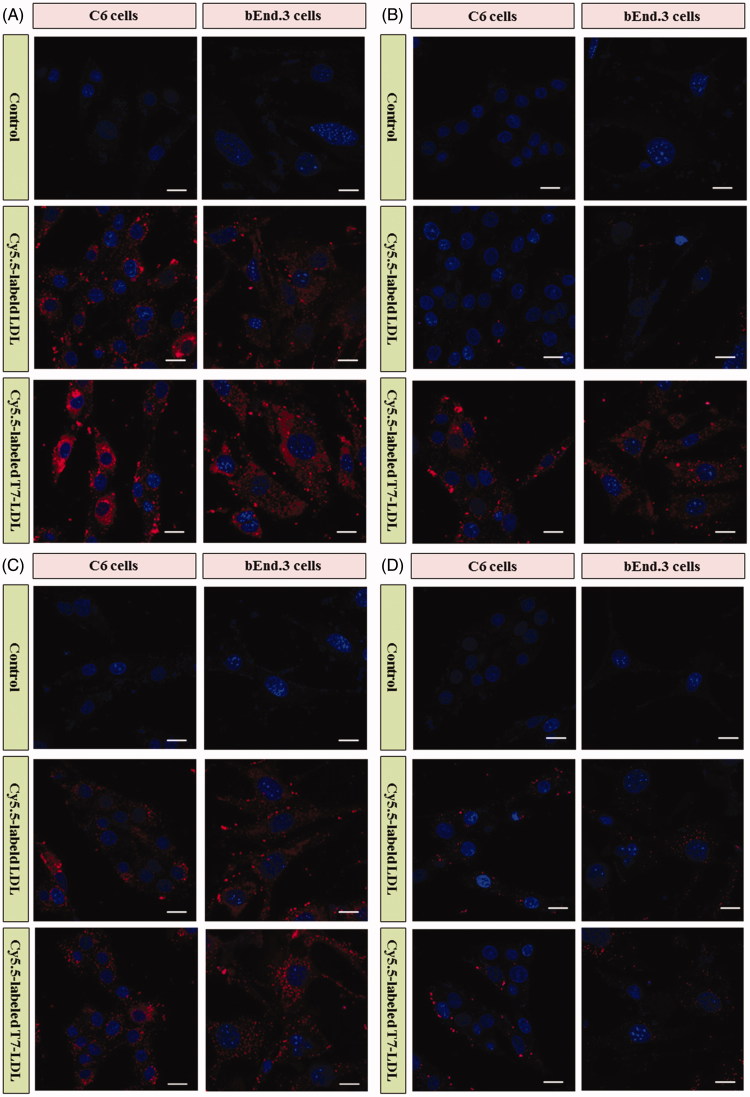
(A) Cellular uptake of various formulations into cells. (B) Cellular uptake of various formulations into cells with excess parent LDL. (C) Cellular uptake of various formulations into cells with excess free T7. (D) Cellular uptake of various formulations into with excess free T7 and parent LDL. Intracellular fluorescence was captured by a confocal laser scanning microscope.

As shown in Figure 3(A), Cy5.5-labeled LDL displayed significant internalization into bEnd.3 cells and C6 cells, while even higher accumulation was observed in the two types of cells incubated with Cy5.5-labeled T7-LDL. The results suggested that BBB cells and glioma cells recognition using dual mediations (TfR and LDLR) were enhanced compared with that of the Cy5.5-labeled LDL (only LDLR-mediated endocytosis). The uptake of Cy5.5-labeled T7-LDL and Cy5.5-labeled LDL was further evaluated after pre-incubation with excess free T7 (1 mg/mL) and/or parent LDL (1 mg/mL) to saturate the cell surface receptors. As shown in Figure 3(B), the binding of two formulations was significantly inhibited by excess parent LDL in the two types of cells, suggesting a role for LDLR mediation. Moreover, the intracellular fluorescence of Cy5.5-labeled LDL even declined to a level similar to that of control. As shown in Figure 3(C), the internalization of Cy5.5-labeled LDL on the two types of cells was not significantly influenced by excess free T7, where very similar fluorescence intensities were exhibited (Figure 3(A)). On the contrary, the binding of Cy5.5-labeled T7-LDL was significantly inhibited by pre-incubation with excess free T7 and the intracellular fluorescence declined to a level similar to that of Cy5.5-labeled LDL. The results demonstrated that when the expression level of TfR on the cell surface was lower, Cy5.5-labeled T7-LDL could not efficiently recognize and bind with the target cell via the T7 motif, and thus, the uptake efficiency of Cy5.5-labeled T7-LDL was suppressed significantly, becoming almost equivalent to that of Cy5.5-labeled LDL. Following pre-incubation with excess free T7 and parent LDL (Figure 3(D) ), a significantly decreased intracellular fluorescence was detected in the two types of cells treated with two formulations, suggesting the deactivation of TfR and LDLR. Taken together, these results indicated that the cellular uptake of T7-LDL was enhanced due to the synergism between TfR and LDLR mediations.

Plasma or serum proteins were reported to associate to nanoparticles and form a new surface named the ‘protein corona’ (PC). Formed PCs can influence the biodistribution, targeting efficacy, and toxicity features of nanoparticles. Recently, a study demonstrated the PC could decrease the binding of T7 with TfR (Zhang et al., 2018). As the influence of serum on the prepared nanoparticles here was not evaluated, the above binding results may not fully and correctly reflect the *in vivo* situations. Therefore, *in vivo* approaches are strongly needed to assess the targeting ability of the designed nanocarriers in this article.

### Optimization of T7 density

The BBB is a major physiological barrier that prevents drugs or drug delivery systems from entering the brain-targeted region. As the T7 was a key factor that will influence the glioma targeting efficiency of T7-LDL greatly (Figure 3), the cellular uptake of Cy5.5-labeled T7-LDL with modifications of different densities of T7 were evaluated in an *in vitro* co-culture model of bEnd.3/C6 cells to guide the formulation optimizing process in this study.

As shown in Figure S3, both Cy5.5-labeled T7-LDL and Cy5.5-labeled LDL exhibited a significant cellular uptake, indicating that these nanocarriers could cross the BBB and target glioma cells. When the peptide/lipid molar ratio was 0.5%, there was no difference between the cellular uptake of Cy5.5-labeled T7-LDL and Cy5.5-labeled LDL (*p* > .05), while the cellular uptake of Cy5.5-labeled T7-LDL was significantly influenced by the increase in peptide/lipid molar ratio from 0.5% to 4%. Cy5.5-labeled T7-LDL could promote uptake in C6 cells after crossing bEnd.3 cells more strongly than Cy5.5-labeled LDL, which could be attributed to peptide functionalized LDL’s targeting ability. This result further supported the data of the cellular uptake in the monolayer cell model (Figure 3(C)). With the further increase of the ratio to 6%, there was no significant difference in uptake compared with Cy5.5-labeled T7-LDL with a 4% ratio. This was possibly caused by the saturation phenomenon of TfR on cells. Limited by the number of receptors and the recycling of endocytosis, receptor-mediated endocytosis is a saturated pathway, which restricts the amount of the T7-LDL that is available for cellular uptake. Considering the above results, the molar ratio of 4% for T7 was selected in next experiments.

As the primary brain capillary endothelial cells (BCECs) maintain many important characteristics of the BBB *in vivo*, the primary cells may be more suitable to be used to establish the BBB models here. However, BCECs are not easy to contracture and hence non-primary cells were chosen here.

### Cytotoxicity

MTT assay was conducted to evaluate the *in vitro* cytotoxicity of blank T7-LDL, blank LDL VCR-loaded T7-LDL and VCR-loaded LDL in C6 cells. As shown in Figure S4, the blank T7-LDL and blank LDL had very little toxic effects against the aforementioned cells, which revealed that T7-LDL and blank LDL were relatively safe. This result also demonstrated that T7 peptide had no effect on cell viability. With increased concentrations of VCR, free VCR could result in obvious anti-proliferative effects to C6 cells, thus proving the anticancer effect on such kind of brain tumors. In addition, the free VCR group displayed the greatest cytotoxicity in C6 cells (IC_50_ values of 21.6 ng/mL). It could be concluded that free VCR could be quickly transported into cells by passive diffusion under *in vitro* conditions. However, VCR-loaded T7-LDL and VCR-loaded LDL underwent possible drug release process after entering the intracellular region. Therefore, free VCR exhibited a stronger inhibitory effect on the proliferation of monolayer C6 cells compared with VCR-loaded nanocarriers. For VCR-loaded T7-LDL and VCR-loaded LDL, the improved cellular uptake led to an anticipated enhanced cytotoxicity effect. This showed that the delivery of VCR by the T7-LDL significantly increased the cytotoxicity (IC50 values of 42.8 ng/mL) when compared with LDL (IC50 values of 75.5 ng/mL). The cytotoxicity studies indicated that the synergistic effect of T7 peptide and parent LDL promoted anti-proliferative activities in C6 cells that over-expressed TfR and LDLR. These results from the cytotoxicities of VCR-loaded T7-LDL and VCR-loaded LDL were consistent with the *in vitro* cellular uptake results shown in Figure 3.

### *In vivo* targeting ability

**Figure 4.**
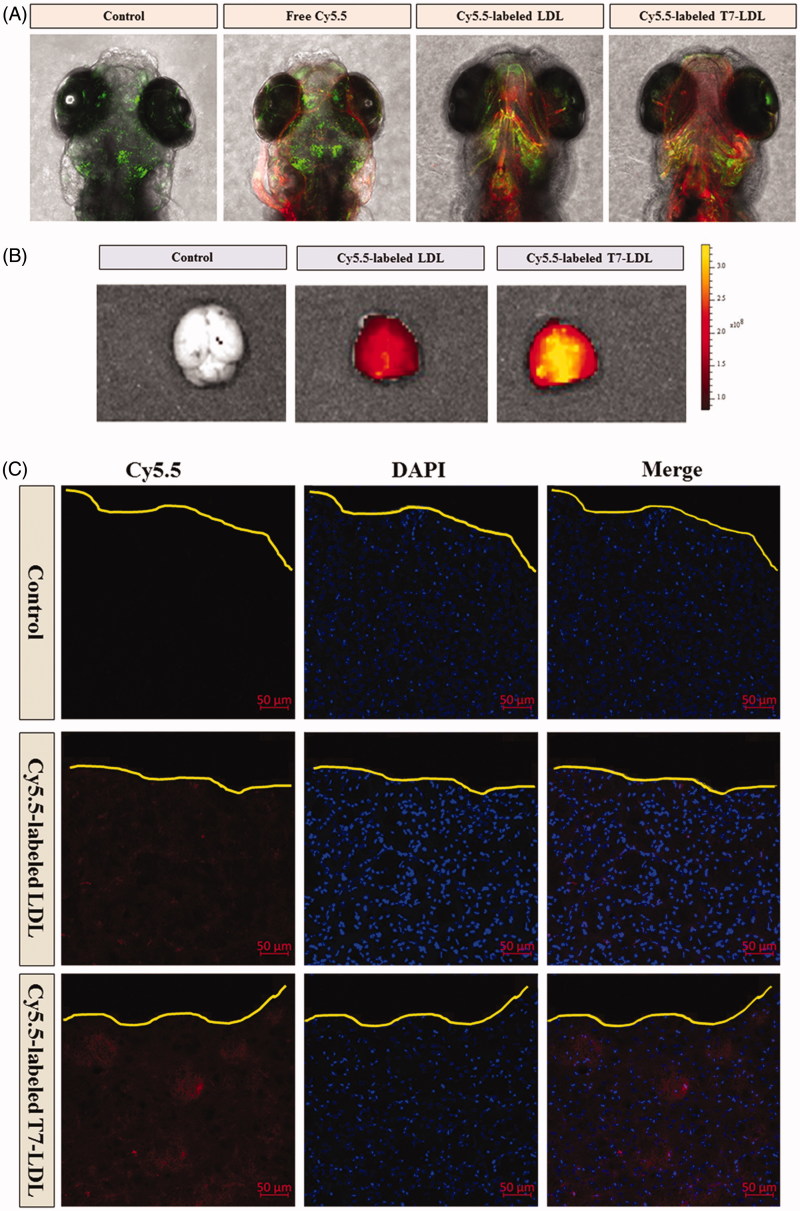
*In vivo* targeting ability. (A) *In vivo* brain imaging in zebrafish. (B) *Ex vivo* fluorescence imaging of the brain. (C) Distribution of Cy5.5 in the brain of mice bearing intracranial C6 glioma determined by a CLSM. The red represents Cy5.5 and the nuclei were stained by DAPI (blue). The yellow line showed the margin of intracranial glioma.

After *in vitro* evaluation of penetrating ability (Figure S3), we examined the ability of Cy5.5-labeled T7-LDL or Cy5.5-labeled LDL to cross the *in vivo* BBB by employing zebrafish which has a similar BBB structure as mammals. The distribution of Cy5.5 fluorescence signals (red) in the brain area and outside of the vessels (green) of zebrafish represents the ability of samples to cross the BBB into brain. This provides a quick, effective, and cost-efficient model for screening the brain-targeted efficacy of nanocarriers *in vivo*. As shown in Figure 4(A), free Cy5.5 (red) was not found in the brain area, but it is only found in the vessels of zebrafish (green) after the injection. The results indicated that free Cy5.5 was confined to the vascular system and did not across the BBB into the brain area in the zebrafish. In contrast, zebrafish injected with Cy5.5-labeled LDL showed a mild fluorescence signal in the brain. Compared with Cy5.5-labeled LDL, Cy5.5-labeled T7-LDL showed a relatively higher accumulation in the brain of zebrafish. The distribution into the brain area and outside of the vessels represents the stronger ability of T7-LDL to delivery drug across the BBB into the brain *in vivo*. We conclude that the T7-LDL could significantly increase the delivery efficiency of brain targeting *in vivo*. These results from zebrafish were consistent with the *in vitro* results shown in Figure S3 (*in vitro* BBB model). The T7-LDL could be used for further study in the treatment of brain cancer *in vivo.*

To estimate the glioma targeting ability of T7-LDL, in the present experiment, the *ex vivo* biodistribution of Cy5.5-labeled T7-LDL or Cy5.5-labeled LDL were imaged by collecting fluorescence signals of the brain and main organs in mice with intracranial C6 glioma. Based on *ex vivo* imaging (Figure 4(B)), there were no signals in the brain of control group, while the brain accumulation was high for the Cy5.5-labeled T7-LDL and Cy5.5-labeled LDL groups. As expected, Cy5.5-labeled T7-LDL exhibited higher accumulation in brain region than did Cy5.5-labeled LDL, suggesting of the importance of the T7 ligand across the BBB to target glioma. *Ex vivo* imaging of the main organs demonstrated limited accumulation in liver in comparison to that in other organs (Figure S5). Compared with Cy5.5-labeled T7-LDL, the liver accumulation was much higher for the Cy5.5-labeled LDL group. This may be because the liver is the major filtering organs in body, nanocarriers are easily trapped in the filter. This is also a shortcoming of T7-LDL to be overcome in the future.

To further evaluate its *in vivo* glioma targeting capability, immunofluorescence assay was conducted after the treatment of various Cy5.5-labeled samples in mice bearing intracranial glioma. As shown in Figure 4(C), no fluorescence was observed in the glioma of the control group. Cy5.5-labeled LDL showed slight accumulation in the glioma region, which was consistent with the results show in Figure 4(A) and (B). It was apparent that significant higher distribution of Cy5.5-labeled T7-LDL was observed in the glioma when compared with Cy5.5-labeled LDL, indicating the precise glioma targeting property of T7-LDL with the modification of T7 ligands. These results are consistent with the *in vitro* results (Figure 3 and Figure S3) and indeed support our hypothesis that the T7-LDL could not only enhance the BBB penetration but also improve glioma targeting. The results again emphasized the advantage of the T7-LDL in glioma targeting delivery.

### *In vivo* therapeutic efficacy

**Figure 5.**
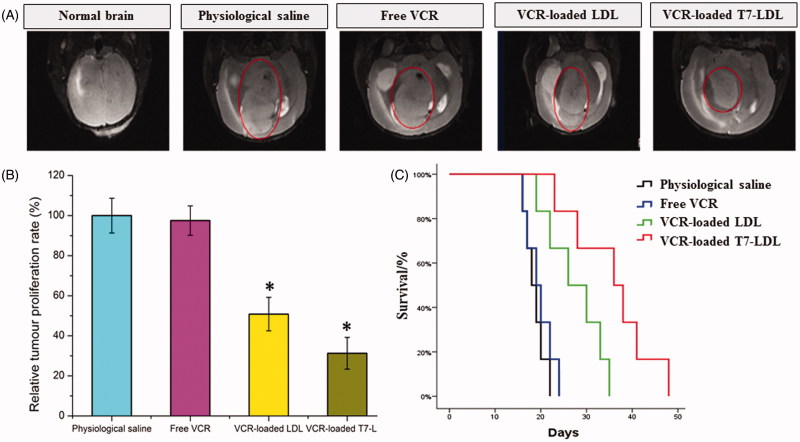
Anticancer efficacy in intracranial C6 glioma-bearing mice. (A) MRI of normal and pathological brains at 16 day after inoculation. (B) Inhibition of the brain glioma volume. (C) Kaplan–Meier survival curves. The data are presented as the means ± SD (*n* = 6). *Indicates *p* < .05.

The *in vivo* antiglioma efficacy was investigated using the mice bearing intracranial C6 glioma. After treatment with the control formulations (physiological saline, free VCR, and VCR-loaded LDL) and VCR-loaded T7-LDL, overall anti-glioma efficacy was observed by MRI for monitoring cancer volume and was confirmed using survival curves. Consistent with the results of glioma distribution (Figure 4(C)), tumor inhibition analysis confirmed the significant glioma targeting effect of VCR-loaded T7-LDL in mice with intracranial C6 glioma. As shown in Figure 5(A), glioma diameter in the brain at day-16 was clearly reduced according to MRI after treated with the VCR-loaded T7-LDL as compared with those after treatment with control formulations, suggestive of VCR-loaded T7-LDL across the BBB and targeting glioma cells. It should be noticed that, the MRI results sometimes were not very accurate due to the moderate correlation between pathology and MRI results (Sekerdag et al., 2017). Relative tumor proliferation rate at day 16 (Figure 5(B)) was 100.00 ± 8.69% for physiological saline, 97.52 ± 7.33% for free VCR (because insufficient drug does reach to the tumor), 51.50 ± 7.73% for VCR-loaded LDL, and 30.26 ± 4.32% for VCR-loaded T7-LDL. These results indicate that the therapeutic efficacy of the VCR-loaded T7-LDL is significantly superior to that of other formulations in intracranial C6 glioma-bearing mice models. Although free VCR could inhibit the growth of monolayer C6 cells more strongly than VCR-loaded nanocarriers (VCR-loaded T7-LDL and VCR-loaded LDL) in the *in vitro* cytotoxicity assay (Figure S4), VCR-loaded nanocarriers could more strongly inhibit the growth of glioma *in vivo* than free VCR, which could be attributed to T7 selectivity and parent LDL delivery.

Histological changes of glioma after different treatments were detected and compared using HE staining. As shown in Figure S6, glioma from mice treated with VCR-loaded T7-LDL displayed abnormal tissue and cells, exhibiting a hypocellular and necrotic zone, where as tumors from mice treated with the other formulations showed a more hypercellular zone and normal nuclear polymorphism. These results indicated that the T7-LDL had a more effective antiglioma activity. The trend observed for the histological analysis was consistent with the results of the biodistribution (Figure 4(A,B,C)) and the antiglioma efficacy *in vivo* (Figure 5(A,B,C)).

The clinical therapeutic benefits are mainly determined based on the quality of life and prolonged survival time of cancer patients. In further investigation of the potential of VCR-loaded LDL in antiglioma therapy *in vivo*, the Kaplan–Meier survival curve of intracranial C6 glioma-bearing mice was used (Figure 5(C)). As expected, treatment with VCR-loaded T7-LDL significantly prolonged the median survival time (36 days), which was 2-, 1.9-, and 1.4-fold higher than that of physiological saline (18 days), free VCR (19 days) and VCR-loaded LDL (26 days), respectively (*p* < .05). This was mainly attributed to the target systemic delivery of T7-LDL, which was demonstrated by brain targeting ability (Figure 4(A)) and glioma targeting ability (Figure 4(B,C)). In addition, the body weight variation of mice was monitored during the experimental period (Figure S7). As shown in Figure S7, More than 12% of weight loss was found in the free VCR group at the end of experimental period. The weight loss of the free drug group was likely due to the non-targeted characteristics and tumor cachexia. While, the smaller weight loss of mice in VCR-loaded T7-LDL group than that of free VCR group during the whole experimental period, indicated the T7 peptide modified LDL nanoparticles reducing unspecific cellular uptake through glioma targeted delivery.

LDL are cholesterol-rich primary cargo vehicles for the delivery of cholesterol in peripheral tissues. However, when LDL, especially oxidized LDL, is excessive, it is prone to cause atherosclerosis. Therefore, LDL is also called ‘bad cholesterol’. For an adult, about 4.5 g of LDL was contented in the whole blood. The drug loading efficiency of VCR-loaded T7-LDL was 3.7% in this article. For the chemotherapy usage of VCR, its dosage was around 2 mg once a week, which is equal to 53.4 mg of VCR-loaded T7-LDL here. It means about 1.2% extra LDL was injected to the body each week (for human), which may have a mild effect on the whole cholesterol metabolism. Of course, if the cholesterol level of patients who receive the VCR-loaded T7-LDL therapy beyond the normal range, the combination usage of statin drugs to lower the cholesterol will be a choice.

## Conclusions

In this study, we designed natural nanoscale drug delivery platform (T7-LDL) achieving systemic glioma-targeted drug delivering by employing T7 peptide. T7-LDL could penetrate BBB and target glioma cells. VCR-loaded T7-LDL could effectively enhance the anti-glioma efficacy *in vitro* and *in vivo*. In recent years, LDL has been used as nanoplatforms for tumor-targeted diagnosis and therapy by many groups. However, most studies have focused on peripheral tumors rather than brain tumors, and to the best of our knowledge, this is the first time the combination of the dual brain glioma-targeting delivery by a specific ligand and parent LDL has been reported. Although the current strategy exhibits an effective glioma-targeting delivery and anti-glioma effect, there remain some shortcomings for the delivery system. For example, the singular properties of proteins are related to their specific structures. The surface modifications of LDL may alter its conformation and affect its activity. As a concept verifying experiment, we did not survey the conformation changes of LDL after modifying by T7 peptide in this study. However, the results of this work revealed that T7-LDL had similar drug loading and releasing profiles, LDLR targeting ability and cytotoxicity as LDL did. For this, the T7-modified LDL nanoparticles did not change its activities that we desired. In a future study, the surface engineering technique for LDL needs to be improved. We will continue to perform *in vitro* and *in vivo* evaluations and further explore the application of T7-LDL in glioma-targeted delivery.
